# Incidence and mortality of hip fracture among the elderly population in South Korea: a population-based study using the National Health Insurance claims data

**DOI:** 10.1186/1471-2458-10-230

**Published:** 2010-05-04

**Authors:** Hye-Young Kang, Kyu-hyeon Yang, Yoon Nam Kim, Seong-hwan Moon, Won-Jung Choi, Dae Ryong Kang, Seong Eun Park

**Affiliations:** 1 Graduate School of Public Health, Institute of Health Services Research, Yonsei University, Seoul, South Korea; 2 Department of Orthopedics, College of Medicine, Yonsei Universtiy, Seoul, South Korea; 3 Graduate School of Public Health, Yonsei University, Seoul, South Korea; 4 Health Insurance Review & Assessment Service, Seoul, South Korea

## Abstract

**Background:**

The lack of epidemiologic information on osteoporotic hip fractures hampers the development of preventive or curative measures against osteoporosis in South Korea. We conducted a population-based study to estimate the annual incidence of hip fractures. Also, we examined factors associated with post-fracture mortality among Korean elderly to evaluate the impact of osteoporosis on our society and to identify high-risk populations.

**Methods:**

The Korean National Health Insurance (NHI) claims database was used to identify the incidence of hip fractures, defined as patients having a claim record with a diagnosis of hip fracture and a hip fracture-related operation during 2003. The 6-month period prior to 2003 was set as a 'window period,' such that patients were defined as incident cases only if their first record of fracture was observed after the window period. Cox's proportional hazards model was used to investigate the relationship between survival time and baseline patient and provider characteristics available from the NHI data.

**Results:**

The age-standardized annual incidence rate of hip fractures requiring operation over 50 years of age was 146.38 per 100,000 women and 61.72 per 100,000 men, yielding a female to male ratio of 2.37. The 1-year mortality was 16.55%, which is 2.85 times higher than the mortality rate for the general population (5.8%) in this age group. The risk of post-fracture mortality at one year is significantly higher for males and for persons having lower socioeconomic status, living in places other than the capital city, not taking anti-osteoporosis pharmacologic therapy following fracture, or receiving fracture-associated operations from more advanced hospitals such as general or tertiary hospitals.

**Conclusion:**

This national epidemiological study will help raise awareness of osteoporotic hip fractures among the elderly population and hopefully motivate public health policy makers to develop effective national prevention strategies against osteoporosis to prevent hip fractures.

## Background

Osteoporosis has become a significant public health problem in recent years, especially with the growth of the elderly population [1]. Hip fracture is the most valid epidemiologic marker of osteoporosis, a major cause of morbidity and mortality in the aging population [1-3], and imposes a considerable burden on patients, payers, and society in terms of premature death, long-term disability, restricted social functioning, cost of care, lost productivity, and informal caregiver time. Previous studies have shown that the incidence rates of hip fractures for Asian people were lower than those for Whites living in Northern Europe and North America [4-8]. However, recent reports have shown an increase in the incidence of hip fractures in Asian populations [4,9,10], and it is projected that 45-70% of global hip fractures will occur in Asia by 2050 due to the growth of the elderly population [1,11].

To reduce the cost of fracture care, it is important to adopt effective prevention strategies to reduce osteoporosis, including dietary supplements such as calcium and vitamin D and pharmacologic therapy. However, the lack of detailed data on hip fracture epidemiology, particularly in developing countries, hampers the development of preventive or curative measures against osteoporosis [12]. Thus, knowledge regarding the incidence of hip fractures and their outcomes, including mortality, is needed to evaluate the impact of osteoporosis on our society, to identify high-risk populations, and to help health care decision-makers allocate scarce resources and prioritize hip fractures along with other important clinical problems.

While the growth of the elderly population (>65 years of age) in South Korea has been as marked as in other Asian countries, rising from 3.8% in 1980 to 7.9% in 2002 [13], epidemiologic information about hip fractures among the elderly in South Korea is scarce. To provide a national estimate on the incidence rate of hip fractures in this population, we need a result derived from a large, population-based database for Korea. The Korean National Health Insurance (NHI) claims database is such a database, containing all medical and prescription drug-claim records for almost 100% of the Korean population. South Korea operates a mandatory universal health insurance system, with a centralized healthcare claims database that provides a unique nationwide source of information on health care resource utilization. We used this database to estimate the annual incidence and 1-year mortality of hip fractures in Koreans over the age of 50. Furthermore, we assessed the patient and provider characteristics associated with post-fracture mortality in order to help identify high-risk patients with poor health outcomes and develop effective treatment strategies after hip fracture incidence. The study results are expected to increase our understanding of the magnitude of the elderly population suffering from hip fractures and to urge public health policy makers to develop effective national prevention strategies for osteoporosis.

## Methods

### Data sources and study population

Records from the Korean NHI claims database from 2002 and 2004 were used to identify patients with hip fractures and to monitor their 1-year mortality. The incidence of hip fractures was defined as patients having a claim record with a diagnosis of hip fracture and a hip fracture-related operation. Since identifying hip fracture cases solely based on diagnosis is likely to cause misclassification problems due to potential miscoding, we combined the information from both diagnosis and surgical records. This conservative approach underestimates the real incidence rate of hip fractures in Korea, but improves the validity of the incidence cases identified from insurance claims data.

All claims records of outpatient visits or hospital admissions of patients aged 50 or older containing a diagnosis of femur fracture (fracture of femur [International Classification of Diseases (ICD)-10 diagnostic code: S72], fracture of the neck of the femur [S72.0, S72.00], pertrochanteric fracture [S72.1, S72.10]) and hip fracture-related operation (open reduction & internal fixation [ICD-10 procedure code: N0601], closed reduction and percutaneous fixation [N0991], total hip replacement [N0711], or hip hemiarthroplasty [N0715]) from January 1 to December 31, 2003 were identified from the NHI claims database. The diagnosis and operation code for hip fracture was selected based on previous epidemiologic studies [1,14] and was confirmed by a panel of four orthopedic clinicians working in four different general hospitals in Korea. The cases having more than one claim record that satisfied the inclusion criteria during 2003 were counted only once.

In general, not all operations are carried out during the first fracture visit or admission. However, most of the operations are performed within a month following the first visit, according to a consultation of orthopedic clinicians in Korea. Thus, we additionally defined the incidence cases as patients who did not have a record of a hip fracture-related operation in the claim record of the initial visit or admission, but had it within a month after the initial visit.

The 6-month period prior to 2003 (i.e., July - December, 2002) was set as a 'window period,' such that patients were defined as incident cases only if their first record of a fracture visit or admission was observed after this 6-month period. Since most of the follow-up treatments for hip fracture are completed within 6 months after the initial fracture, we assumed that the absence of any claims record with a diagnosis of hip fracture and hip-fracture-related operation in the previous 6 months assured that the fracture was a new case.

NHI claims data were merged with national mortality data provided by the National Statistical Office to determine the survival status of individual patients at the 12 months following the incidence.

### Data analysis

Age- and sex-specific annual incidence rates of hip fractures per 100,000 inhabitants were calculated and presented in 5-year age intervals. The number of inhabitants in South Korea, as of 2003, was obtained from the Korean National Statistical Office. The age-standardized incidence rate was computed using the Korea Census data of 2000. Age- and sex-specific 1-year mortality rates of hip fractures were calculated and presented in 5-year age intervals. To examine the difference in the incidence and mortality of hip fractures according to gender, the gender ratio of incidence and 1-year mortality rate was computed for each 5-year age stratum.

Cox's proportional hazards model was used to investigate the relationship between survival time and baseline patient and provider characteristics. The independent variables were age, gender, type of fracture, type of operation, baseline health status, residence area, type of health insurance, provision of anti-osteoporosis medication therapy after fracture, and type of hospital providing operation.

## Results

During the 1-year observation period from January through December 2003, there were 9,817 incidents of hip fractures in persons aged 50 and older in South Korea: 6,892 females (70.2%) and 2,925 males (29.8%). The mean (± SD) and the median age were 74.92 (± 9.13) and 76 years old, respectively. The mean age was younger in men (72.10 ± 9.47 years) than in women (76.11 ± 8.72 years). The age-standardized annual incidence rate of hip fractures was 104.06 per 100,000; 146.38 per 100,000 women and 61.72 per 100,000 men, yielding a female/male ratio of 2.37 (Table 1).

**Table 1:** Sex- and age-specific annual incidence rates of hip fractures in South Korea in 2003

	Total			Female			Male			
		
Age (years)	No. population 2003	**No. hip fx**.	Incidence per 100,000	No. popl 2003	**No. hip fx**.	Incid. Per 100,000	No. popl 2003	**No. hip fx**.	Incid. Per 100,000	**F/M ratio of incid**.
50-54	2,567,149	173	6.74	1,274,375	69	5.41	1,292,774	104	8.04	**0.67**
55-59	2,104,791	403	19.15	1,061,917	205	19.30	1,042,874	198	18.99	**1.02**
60-64	1,935,661	824	42.57	1,023,028	449	43.89	912,633	375	41.09	**1.07**
65-69	1,594,318	1,311	82.23	882,721	817	92.55	711,597	494	69.42	**1.33**
70-74	1,099,247	1,790	162.84	665,698	1,245	187.02	433,549	545	125.71	**1.49**
75-79	683,904	1,982	289.81	441,732	1470	332.78	242,172	512	211.42	**1.57**
80-84	380,112	1,888	496.70	259,291	1456	561.53	120,821	432	357.55	**1.57**
85+	210,537	1,446	686.82	159,330	1181	741.23	51,207	265	517.51	**1.43**

Overall (50+)	10,575,719	9,817	**92.83**	5,768,092	6,892	**119.48**	4,807,627	2,925	**60.84**	**1.96**

Age-standardized rates*			**104.06**			**146.38**			**61.72**	**2.37**

*Age-standardized incidence rate was computed using Korea Census data of 2000.

**Figure 1:**
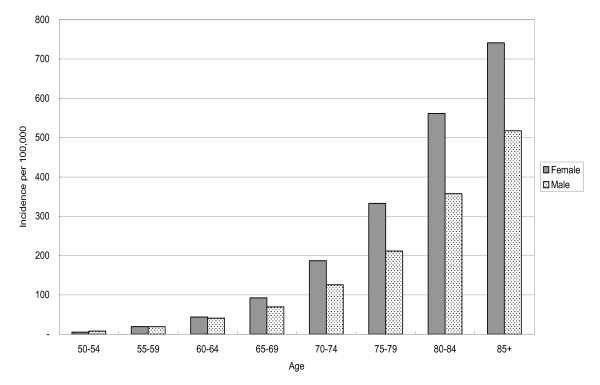
**Comparison of age-specific incidence rates of hip fracture according to gender**.

The incidence rate of hip fractures increased with increasing age, from 6.74 per 100,000 for the youngest group of 50-54 year olds to 686.82 per 100,000 for the oldest group of 85 years and older. This increase followed an exponential relationship after age 65 for both genders (Figure 1). About 34.0% of all hip fractures occurred in patients aged 80 years and older, although this age group comprised only 5.6% of the total population over 50.

The gender ratio of the female to male incidence rate was computed for the 5-year age stratum. The ratio for the youngest 5-year group (50-54 years old) was smaller than 1, indicating a higher incidence rate among males than among females, probably due to occupation. However, this ratio increased with increasing age and was greater than 1 for all groups older than or equal to 55 years old.

Of 9,817 patients, 1,625 died during the first year post-fracture, yielding a mortality rate of 16.55% (Table 2). The mortality rate increased with increasing age from less than 10% of patients aged younger than 65 to more than 20% of those over 80 years old. Male patients consistently showed a higher mortality rate than female patients in all age categories except the youngest, with the ratio of female to male mortality ranging from 0.48 to 0.72, compared with 3.77 in the youngest group.

**Table 2:** Sex- and age-specific post-hip fracture mortality rate at 1 year

	Total			Female			Male			
		
Age (years)	No. hip Fx. 2003	No. mortality	Mortality rate (%)	No. hip Fx. 2003	No. mortality	Mortality rate (%)	No. hip Fx. 2003	No. mortality	Mortality rate (%)	F/M ratio of mortality rate
50-54	173	14	8.09	69	10	14.49	104	4	3.85	**3.77**
55-59	403	21	5.21	205	7	3.41	198	14	7.07	**0.48**
60-64	824	76	9.22	449	32	7.13	375	44	11.73	**0.61**
65-69	1,311	150	11.44	817	78	9.55	494	72	14.57	**0.66**
70-74	1,790	216	12.07	1,245	125	10.04	545	91	16.70	**0.60**
75-79	1,982	347	17.51	1470	224	15.24	512	123	24.02	**0.63**
80-84	1,888	413	21.88	1456	293	20.12	432	120	27.78	**0.72**
85+	1,446	388	26.83	1181	296	25.06	265	92	34.72	**0.72**

Overall (50+)	9,817	1,625	16.55	6,892	1,065	15.45	2,925	560	19.15	**0.81**

Patient and provider characteristics available from the NHI claims data were examined for their association with 1-year post-fracture mortality using the multivariate Cox-proportional hazard model (Table 3). While other factors were held constant in the multivariate model, gender and age remained statistically significant factors affecting patient survival after hip fracture. As compared to the patients residing in Seoul, the capital city of South Korea, those living in other parts of South Korea had a significantly higher risk of death following fracture, which showed a hazard ratio ranging from 1.291 (95% confidence interval (CI): 1.120-1.488) for those in metropolitan cities to 1.656 (95% CI: 1.344-2.039) for those in non-city areas.

**Table 3:** Multivariate survival analysis: Patient and provider characteristics associated with 1-year mortality after hip fracture

	Variables	N (%)	Hazard ratio	95% confidence interval
Gender	Female	6,892 (70.2)	1.000	
	Male	2,925 (29.8)	1.400	(1.260-1.557)
Age (years)	50-59	576 (5.9)	1.000	
	60-69	2,135 (21.7)	1.787	(1.250-2.553)
	70-79	3,772 (38.4)	2.879	(2.041-4.062)
	80-89	2,923 (29.8)	4.742	(3.361-6.691)
	≥ 90	411 (4.2)	9.273	(6.387-13.463)
Residence	Seoul (Capital city)	2,388 (24.3)	1.000	
	Metropolitan city	2,584 (26.3)	1.291	(1.120-1.488)
	Small city	4,133 (42.1)	1.214	(1.058-1.392)
	Others (none-city area)	712 (7.3)	1.656	(1.344-2.039)
Health insurance	National Health Insurance program	8,514 (86.7)	1.000	
	National Medical Aid program	1,303 (13.3)	1.300	(1.136-1.488)
Baseline	0	4,742 (48.3)	1.000	
Charlson	1	4,038 (41.1)	1.339	(1.198-1.496)
Index (before hip fracture)	2	938 (9.6)	2.625	(2.264-3.042)
	3, 6	99 (1.0)	5.693	(4.249-7.627)
Type of hip fracture	Neck fracture	5,435 (55.4)	1.000	
	Petrochanteric fracture.	3,733 (38.0)	1.090	(0.966-1.230)
	Neck + Petrochanteric fracture.	649 (6.6)	1.031	(0.843-1.261)
Type of operation	Total hip replacement (ICD 10^th ^procedure code: N0711)	4,574 (46.6)	1.000	
	Open reduction/internal fixation (N0601)	4,592 (46.8)	0.963	(0.855-1.084)
	Closed reduction/percutaneous fixation (N0991)	397 (4.0)	0.958	(0.720-1.274)
	Hip hemiarthroplasty (N0715)	216 (2.2)	0.793	(0.536-1.174)
	Total hip replacement & Open reduction (N0711 + N0601)	38 (0.4)	1.213	(0.575-2.561)
Treatment*	No	7,761 (79.1)	1.000	
	Yes	2,056 (20.9)	0.576	(0.499-0.666)
Type of hospital providing operation	Tertiary hospital	2,714 (27.6)	1.000	
	General hospital	4,649 (47.4)	0.900	(0.796-1.018)
	Hospital	1,901 (19.4)	0.798	(0.680-0.937)
	Clinic	553 (5.6)	0.746	(0.573-0.972)

* Treatment was defined as whether the patient took at least one prescription of anti-osteoporosis medicine after hip fracture.

To examine the impact of socioeconomic status on the mortality of hip fractures, we compared the mortality between elderly enrolled in the two tiers of the Korean universal health insurance system: the Health Insurance (HI) and the Medical Aid (MA) program. The HI is a wage-based, contributory insurance program covering about 97% of the population, whereas the MA is a government-subsidized, public assistance program for the poor and the medically indigent. The NHI claims data include records for both the HI and the MA program populations. The survival analysis showed that patients enrolled in the MA program had a 30% higher chance of death during the year following fracture than those in the HI program (hazard ratio: 1.300, 95% CI: 1.136-1.488).

As a proxy measure for patients' baseline health status, a Charlson comorbidity index [15] was assigned to each study subject based on the diagnosis shown in the NHI claims record during the study period prior to hip fracture. Charlson et al. developed a scoring system for comorbid illness, such that each comorbid condition was assigned a whole number integer ranging from 1 to 6, which was proportional to the relative risk of 1-year mortality associated with that disease [16]. Not surprisingly, those who had had severe comorbid conditions before they had the fracture outbreak were 1.339 (for those with Charlson Index of 1), 2.625 (Index of 2), or 5.693 (Index of 3 or 6) times more likely to die as compared to those without any of the Charlson Index conditions.

The risk of mortality following fracture was not significantly different according to the type of hip fracture or operation. The likelihood of dying among those with claims records showing at least one reimbursed prescription for anti-osteoporosis drugs (alendronate, etidronate, raloxifen, risedronate, and salcatonin) following hip fracture was 42.4% lower than those without such claims records.

As the level and the size of the hospital in which the patients received their operation decreased, so did the chance of death. The hazard ratios of 1-year mortality, with tertiary hospitals as a reference group, were 0.798 (95% CI: 0.680-0.937) for hospitals and 0.746 for clinics (95% CI: 0.573-0.972).

## Discussion

This is the first nation-wide, population-based, epidemiologic study of hip fractures in Korea and one of the few studies worldwide reporting the incidence of hip fractures based on national health insurance claims data. The age-standardized annual incidence rate for the entire population of Korea over 50 years of age in 2003 was estimated as 104.06 per 100,000 inhabitants, which is about 27.8% lower than that estimated in an earlier study conducted on inhabitants residing in selected regions of Korea in 2001 (133 per 100,000) [17]. The difference in the estimated incidence rates between the two studies can be explained by the difference in the dataset, methods used in each study to identify incidence cases, and the population characteristics in the study regions. In the earlier study, hip fracture cases admitted to selected hospitals in Gwangju City and Chonnam Province were identified on the basis of medical records and radiographs. Among the identified incidence cases in that study, 88% underwent surgery. By applying this operation rate to the incidence rate estimated by our study, the total number of incidence cases with and without operation was estimated as 118.3 per 100,000 (= 104.6/0.88), thereby reducing the gap in the incidence estimates between the two studies from 27.8% to 12.4%.

After standardization according to the age distribution of the US white population in 1990 [18], the incidence rates of hip fractures in Korea were 173 per 100,000 in women and 91 per 100,000 in men. Interestingly, the standardized incidence rates of both genders were substantially lower than those of most of the Asian countries including Taiwan (505 per 100,000 in women and 225 per 100,000 in men), Hong Kong (459 and 180 per 100,000), Singapore (442 and 164 per 100,000), and Thailand (269 and 114 per 100,000) [9].

The risk of 1-year mortality following hip fracture was 16.55%, which is about 2.85 times higher than the mortality rate of the general population in South Korea over 50 years of age. Despite the heterogeneity of the study population in terms of age and ethnicity, and the potential discrepancy in the quality of follow-up care after the fracture, the post-fracture mortality at 1 year observed in other countries is similar to that in Korea. A recent U.S. study has reported that about 11.5% of 950 patients over age 65 admitted to selected hospitals with a hip fracture between 1987 and 1997 died after 1 year [19]. The 1-year mortality rate was 15.7% among 541 fracture patients over 60 years of age admitted to one university hospital in Greece between 1989 and 1992 [20]. In a population-based, case-control study in Sweden, about 10.6% of 2,245 women aged 50-81 years admitted with a hip fracture died within 1 year [21]. The comparison with other Asian countries also showed similar results. The 1-year mortality of patients with surgically treated hip fractures at a hospital in Taiwan between 1998 and 2006 was about 14.0% [22]. A Japanese study by Muraki et al. reported that the 1-year mortality rate following hip fracture was 11.5% [23].

Osteoporosis is recognized as a problem predominantly in elderly women [24,25]. Consequently, women have higher incidence rates of hip fracture than men. In Korea, the female to male ratio of the age-standardized incidence rates of hip fractures for those aged 50 and older is 2.37. This ratio is comparable to western countries such as Sweden (2.2, 1987-1991), Norway (2.3, 1983-1984), Australia (2.7, 1989-1990), and in U.S. whites (2.7, 1983-1984), and in some Asian countries such as Thailand (2.3, 1997-1998), Hong Kong (2.5, 1997-1998), and Malaysia (2.4, 1997-1998) [3]. Hip fracture rates were higher in women of all age groups except for those under 55 years. This pattern is commonly observed in populations of other countries, although the starting age at which women show a higher hip fracture rate varies from country to county: 60 in Taiwan and Japan [4,9], but mostly 55 or 50 in other countries such as Mexico, Argentina, Iran, and Morocco [1-3,5].

Although women showed consistently higher incidence rates of hip fractures than men, the high incidence rates among older men is worthy of attention. After age 75, Korean men experienced hip fractures at a rate of 211.42 to 517.51 per 100,000 inhabitants. This rate is similar to that observed in other Asian countries, such as Japan (209.0 to 780 per 100,000), Hong Kong (404 to 1,639), Singapore (611), Malaysia (320), and Thailand (227 to 727) [4,6]. The increased incidence with increasing age in both genders confirms the equal vulnerability of both genders to the aging process. Thus, preventive strategies are important for both genders.

It is interesting to observe higher post-fracture mortality in Korean males than in females. Gender differences in post-hip fracture mortality have been reported in earlier studies showing that men were about twice as likely as women to die during the first years after hip fracture [26-28]. The observed difference between men and women could be explained by under treatment of osteoporosis [28] and higher infection rates in men following hip fracture [26,27].

Low socioeconomic status among the elderly population seems to be associated with an increased risk of fracture-caused mortality. The adjusted hazard ratio of post-fracture mortality was significantly higher among those enrolled in the MA program than among those in the HI program. Patients with low socioeconomic status, in general, have poorer health status and therefore tend to have poorer health outcomes from the same conditions as compared to those with higher socioeconomic status [29,30]. Thus, more effective public health strategies to treat osteoporosis and to prevent fracture incidence should be implemented that target the indigent elderly population in Korea.

The regional variation in fracture-related mortality among Korean elderly is noteworthy. Those living in places other than the capital city consistently showed a higher risk of death during the first year after hip fracture, with a minimum of 21.4% or a maximum of 65.6% additional chance of death. This discrepancy may be due to the lack of access to health care providers or the poor quality of health care provided in non-city or small city areas. Further investigation is necessary to figure out the reasons for this regional discrepancy and therefore to reduce this discrepancy.

From our analysis, patients receiving medication to treat osteoporosis after fracture were 42.4% less likely to die within the year following fracture. The association between reduced mortality and post-fracture use of anti-osteoporosis drugs in elderly hip fracture patients has been addressed in earlier studies. A prospective analysis from Finland has reported a 43% reduction in deaths at 36 months following hip fracture in females who used prescribed calcium plus vitamin D supplementation concomitantly with anti-osteoporosis drugs [31]. Also, an observational study from Canada has shown that mortality is significantly lower in the group treated with anti-osteoporosis drugs than in the untreated group following hip fracture [32]. Because elderly people with prior fractures are at a higher risk for future fractures [33], clinical practice guidelines recommend the initiation of pharmacologic treatment of osteoporosis after the first fracture [34]. However, under-treatment of osteoporosis [35,36] and low adherence to oral anti-osteoporosis treatment following hip fracture [37] is commonly observed. In our study, only 20.9% of the fractured patients received anti-osteoporosis drugs following hip fracture. Thus, a more aggressive practice of diagnosing and providing medication therapy for osteoporosis seems to be an urgent need in Korea.

Interestingly, patients that underwent an operation for hip fracture from hospitals or clinics tend to have a better chance of survival than those from tertiary hospitals. Although our multivariate model tried to include as many patient characteristic variables as possible from the NHI data, we could not comprehensively control for hip fracture severity and patient health conditions due to the lack of clinical information available from the claims records. Thus, it cannot be concluded that the quality of care and patient outcomes after hip fracture treatment are better among hospitals and clinics than among tertiary hospitals in Korea. It might be possible that patients with more severe conditions underwent hip fracture repairs from larger and more advanced hospitals (i.e., tertiary or general hospitals) and therefore the prognosis after surgery tended to be poorer. A more thorough adjustment for patient baseline disease severity is necessary to make a concrete conclusion about this finding.

Since a hip fracture is a severe condition, most of the care episodes are initiated with hospital admission and most prior studies have relied on hospitalized hip fractures to identify incident cases [14,17]. However, if we restrict the study sample to include hospitalized fractures only, we will underestimate incidence rates by excluding less severe cases that are initiated with office visits. To overcome this problem, the present study defined incident cases either by initial admission or a visit containing a diagnosis of hip fracture. However, while most hospital admissions coincide with an acute new episode of illness, it is difficult for outpatient visits to determine whether the case is a new episode or part of a post-episode course. In addition, the accuracy of diagnostic codes among the claims data for office visits is lower than that of hospital admission [38,39]. Thus, to improve the accuracy of identifying incidence cases based on initial fracture visits, we excluded patients with a claims record with a hip fracture during the 6 months prior to the initial visit. Furthermore, to confirm the hip fracture cases, hip fracture-related operation codes were used in addition to the diagnostic codes.

Several methodological issues arise from the studies using administrative claims data. First, not all patients with fractures have access to hospitals. The incidence based on insurance claims records would be underestimated if many hip fractures in Korea were not diagnosed or treated in health care institutions. However, due to the emergent character of hip fractures, it is believed that virtually all hip fractures present to health services [3]. Thus, the incidence of hip fractures identified from claims records is considered to be close to the actual incidence.

Second, the reliance on ICD-10 diagnostic codes to identify incident fractures may cause misclassification of incident hip fractures due to the inherent nature of claims data such as voluntary or non-voluntary miscoding behavior. However, a recent validation study for the diagnostic codes of the NHI claims database in Korea has alleviated this concern. It has been revealed that about 70% of primary, secondary, or tertiary diagnosis codes from NHI claims records coincide with those from medical records. In addition, the accuracy of diagnosis codes tended to be higher for claims from hospital admissions compared with office visits, and for claims of severe conditions compared with mild conditions [38,39].

## Conclusion

In conclusion, our results indicate a high incidence and mortality rate of hip fractures for both men and women living in South Korea. These high incidence rates for both genders are sentinel signals of the impact of hip fractures in Korea, in terms of high mortality, morbidity, and health care costs for treatment. It is our hope that this national epidemiological study will help raise the awareness of hip fractures among the elderly population and support the practice of aggressive diagnosis and the adoption of effective treatment options for osteoporosis.

## List of abbreviations

CI: confidence interval; HI: Health Insurance; MA: Medical Aid; NHI: National Health Insurance.

## Competing interests

The authors declare that they have no competing interests.

## Authors' contributions

HYK developed the study design, interpretted the data, and prepared the manuscript draft. KHY and SHM consulted clinical matters regarding osteoporosis, hip fractures, and their treatment, and helped to develop the implication of the study findings. YNK and WJC performed the statistical analysis. DRK participated in its design and provided statistical advice. SEP helped to draft the manuscript and performed the necessary reference searches. All authors have read and approved the final manuscript.

## Pre-publication history

The pre-publication history for this paper can be accessed here:

http://www.biomedcentral.com/1471-2458/10/230/prepub
